# Association of telomere length with cognitive function and dementia: A cross-sectional NHANES analysis and 2-sample Mendelian randomization study

**DOI:** 10.1097/MD.0000000000048217

**Published:** 2026-04-10

**Authors:** Yuhang Shen, Jingdong Zhou, Bing Sun, Li Geng

**Affiliations:** a Department of Vascular Surgery, The Second Hospital of Jilin University, Changchun, China; b Department of Neurology, Changchun Central Hospital, Changchun, China.

**Keywords:** cognitive function, dementia, Mendelian randomization study, NHANES, telomere length

## Abstract

This study aimed to investigate the association between telomere length, cognitive function, and dementia, and to assess causality using Mendelian randomization (MR). This study included 1815 participants aged over 60 from the National Health and Nutrition Examination Survey (NHANES) conducted between 1999 and 2002. Cognitive function was assessed using the digit symbol substitution test score, and telomere length was quantified as the mean leukocyte telomere length (telomere/single-copy gene) measured by quantitative polymerase chain reaction. Weighted multivariate linear regression was applied to examine the relationship between cognitive function and telomere length. Additionally, a 2-sample MR approach was employed to explore the genetic causal relationship between telomere length and dementia, and its subtypes, using summary-level genome-wide association studies data derived primarily from individuals of European ancestry. The primary analysis utilized inverse variance weighting, and a series of sensitivity analyses were conducted to ensure robustness of the results. In the cross-sectional observational analysis, longer telomere length was positively associated with higher digit symbol substitution test scores after full adjustment (T2 vs T1: β = 2.1, 95% confidence interval [CI]: 0.35–3.9, *P* = .022; T3 vs T1: β = 2.5, 95% CI: 0.69–4.3, *P* < .001). In the MR analysis, genetically predicted longer telomere length was causally associated with a reduced risk of all-cause dementia (odds ratio [OR] per 1-standard deviation increase: 0.90, 95% CI: 0.83–0.98, *P* = .017), Alzheimer’s disease (OR: 0.87, 95% CI: 0.76–0.99, *P* = .036), and vascular dementia (OR: 0.80, 95% CI: 0.65–0.97, *P* = .026). However, no causal association was found with frontotemporal dementia or Parkinson’s disease. Observational and genetic evidence jointly suggest that longer telomere length is associated with better cognitive function and may play a protective causal role against major forms of dementia.

## 1. Introduction

Normal cognitive decline, such as slowed thinking and reduced concentration, is a natural part of aging. In contrast, abnormal decline involves more severe and widespread deficits across multiple domains, including memory, attention, language comprehension, and executive function. These impairments can disrupt occupational, familial, and social functioning, imposing a substantial burden on affected individuals and society as a whole.^[1,2]^ Mild cognitive impairment (MCI) is often considered an intermediate state between normal aging and dementia, and may progress over time.^[3,4]^ Dementia arises from various causes, including Alzheimer’s disease (AD), vascular dementia (VD), frontotemporal dementia (FTD), and Parkinson’s disease (PD), with AD accounting for approximately two-thirds of all cases. The global prevalence of dementia among adults over 65 years of age is estimated at 7%, with even higher rates (8–10%) reported in developed countries.^[5]^ With population aging, MCI and dementia, have become pressing public health concerns, and projections suggest that up to 120 million people worldwide may be affected by 2050.^[5,6]^ Identifying modifiable risk factors for MCI and dementia is therefore essential for prevention and public health planning.

Telomeres are ribonucleoprotein complexes located at the ends of chromosomes that play a critical role in maintaining chromosomal integrity during replication.^[7,8]^ Telomere length gradually shortens in most somatic cells over time, primarily due to chronic inflammation and oxidative stress.^[9]^ Numerous studies have demonstrated associations between shorter telomere and a variety of age-related diseases, including cardiovascular disease,^[10]^ cancer,^[11]^ and neurological disorders.^[12]^

However, evidence linking telomere length and cognitive function or dementia have been inconsistent. While some studies have reported no significant differences in telomere length between individuals with and without dementia or cognitive impairment,^[13,14]^ others have suggested that shorter telomeres are associated with increased risk.^[15-17]^ These discrepancies may reflect methodological limitations, such as small sample sizes, cross-sectional designs, and inadequate control for confounding variables. Moreover, most prior studies have relied on observational data, making it difficult to establish whether the observed associations are causal.

To overcome these limitations, different approaches are needed. Large-scale population surveys such as the National Health and Nutrition Examination Survey (NHANES) enable the investigation of cross-sectional associations between telomere length and cognitive function in a nationally representative sample. However, observational analyses remain vulnerable to confounding and reverse causation. Mendelian randomization (MR), which uses genetic variants as instrumental variables (IVs) for telomere length, can strengthen causal inference by reducing these biases. By combining NHANES data with MR analysis, we aimed to evaluate both the population-level association of telomere length with cognitive function and potential causal effect of telomere length on dementia and its subtypes. Accordingly, we hypothesized that leukocyte telomere length would show a positive association with cognitive function in the NHANES cross-sectional analysis, and that genetically predicted longer telomere length would causally reduce the risk of dementia in the MR framework.

## 2. Materials and methods

### 2.1. Overall study design

This study was conducted in 2 phases. In phase 1, data from the 1999 to 2002 NHANES date were used to assess the association between telomere length and cognitive function through multivariate linear regression analysis. In phase 2, MR was employed using summary-level data from genome-wide association studies to evaluate the potential causal relationship between telomere length and dementia. Additionally, we examined whether telomere length causally influenced specific subtypes of dementia.

### 2.2. Cross-sectional study

#### 2.2.1. Data sources

**Figure 1.**
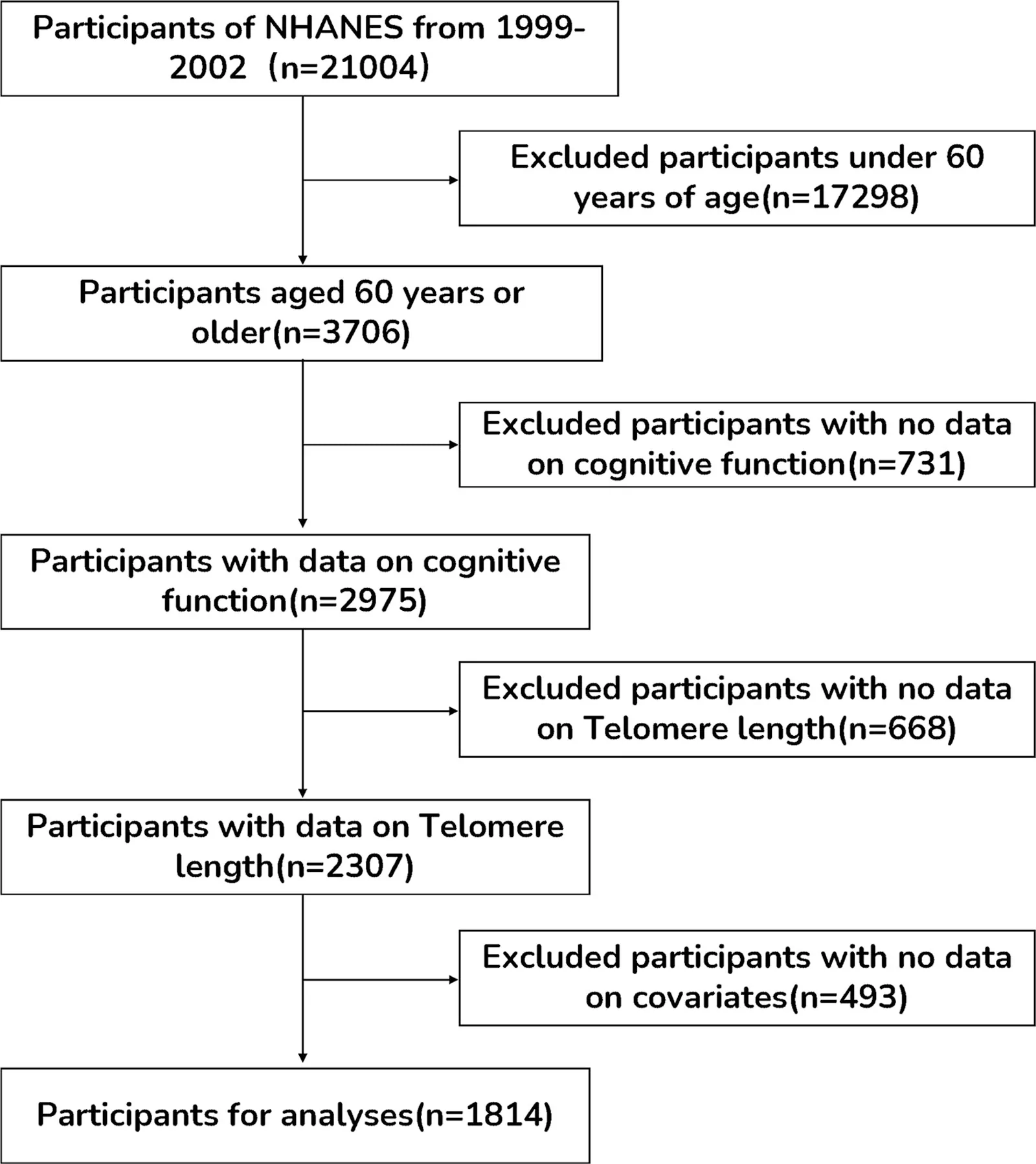
Flowchart of participants selection for the cross-sectional analysis using the National Health and Nutrition Examination Survey 1999–2002 data.

This study utilized data from the 1999 to 2002 NHANES, which covered both telomere length and cognitive function. Participants aged 60 years and older (n = 3706) were initially considered. After excluding individuals with missing data on cognitive function questionnaires (n = 731), telomere length measurements (n = 668), and relevant covariates (n = 493), a final sample of 1814 participants was included in the analysis. The detailed selection process is illustrated in Figure 1. The study protocol was approved by the Ethics Review Board of the National Center for Health Statistics, and written informed consent was obtained from all participants.

#### 2.2.2. Telomere length

Telomere length was the primary variable of interest in this study. As telomeres naturally shorten with age, their measurement provides insights into biological aging. Telomere length was measured using quantitative polymerase chain reaction in the laboratory of Dr Elizabeth Blackburn at the University of California, San Francisco, and was expressed as the telomere/single-copy gene relative to a standard reference deoxyribonucleic acid (DNA) sample.^[18]^

Each sample was assayed in triplicate on 3 separate days. For each run, samples were tested in duplicate wells, yielding 6 measurements per sample. Assays were conducted in batches of 3 plates, with no 2 plates grouped more than once. Each assay plate included 96 control wells containing 8 control DNA samples.

Quality control procedures were rigorously applied. Assay runs were excluded if they had 8 or more invalid control wells (<1% of runs). To normalize between-run variability, control DNA values were used. Runs were also excluded if more than 4 control DNA values fell outside 2.5 standard deviations of the overall mean (<6% of runs). For each sample, potential outliers among the 6 replicate values were identified and excluded (<2% of samples). The final telomere/single-copy gene for each sample was calculated by averaging the valid values. The inter-assay coefficient of variation was 6.5%.

#### 2.2.3. Assessment of cognitive function

Cognitive function was evaluated using the digit symbol substitution test (DSST), a well-validated and widely used neuropsychological assessment tool.^[19]^ The DSST measures multiple cognitive domains, including processing speed, sustained attention, visuospatial abilities, associative learning, and working memory. Participants are required to match symbols with corresponding numbers based on a key, under timed conditions. Scoring is performed using a standardized template, with a maximum possible score of 133 points. Higher scores reflect better cognitive performance, as they indicate a greater number of correctly completed symbol-number pairings within the allotted time.^[20]^

#### 2.2.4. Other covariates

The selection of covariates in this study follows the principle of “prior knowledge driven” to identify and control for potential confounders related to exposure or outcomes. The selected covariates have been widely applicable in previous studies.^[21–23]^ Specifically, the following covariates were included in the analysis as potential confounders in the relationship between telomere length and cognitive function: age, sex, ethnicity, education level, income, physical activity, smoking status, alcohol status, underlying medical conditions, and body mass index.

Participants were categorized into 2 age groups: 60 to 69 years and ≥70 years. Ethnicity was classified as non-Hispanic White, non-Hispanic Black, Mexican American, Other Hispanic, and Other Race. The level of education was grouped as “High school or less” and “College graduate or above.” Income was assessed using the poverty income ratio and categorized into 4 levels: < 1.00 (below the poverty line), 1.00–1.99, 2.00–3.99, and ≥4.00 (highest income group).

Physical activity was assessed based on average daily activity level and classified into 4 categories: very low, low, medium, and high. Smoking status was divided into never smokers (fewer than 100 cigarettes smoked in a lifetime), former smokers (more than 100 cigarettes smoked in a lifetime but currently not smoking), and current smokers (more than 100 cigarettes smoked in a lifetime and currently smoking). Alcohol status was dichotomized as “No” (fewer than 12 drinks/yr) and “Yes” (12 or more drinks/yr).

Underlying medical conditions were self-reported and included hypertension, diabetes, congestive heart failure, stroke, and cancer. Body mass index was categorized as underweight (<18.5 kg/m^2^), normal (18.5–24.9 kg/m^2^), overweight (25–29.9 kg/m^2^), and obese (≥30.0 kg/m^2^).

#### 2.2.5. Statistical analysis

Data from the 1990 to 2000 and 2001 to 2002 survey cycles were combined. Following NHANES analytical guidelines, 4-year sampling weights were calculated and applied to all analyses to account for unequal probabilities of selection and non-response. The complex survey design, including strata and primary sampling units, was incorporated into the analysis. Continuous variables were expressed as weighted means (standard errors) and categorical variables are weighted proportions (%). Group differences in participant characteristics across DSST performance categories were examined using weighted chi-square tests for categorical variables and weighted t-tests for continuous variables. Participants were divided into tertiles according to telomere length. Weighted multiple linear regression models were used to examine the relationship between telomere length (continuous and tertiles) and DSST scores, adjusting for potential covariates described above. Restricted cubic spline models were applied to explore potential non-liner dose-response relationships between telomere length and DSST scores. All statistical analyses were performed in R (version 4.4.3; R Foundation for Statistical Computing, Vienna, Austria). Statistical significance was defined as a 2-sided *P* < .05.

### 2.3. Mendelian randomization analysis

#### 2.3.1. Basic settings

**Figure 2.**
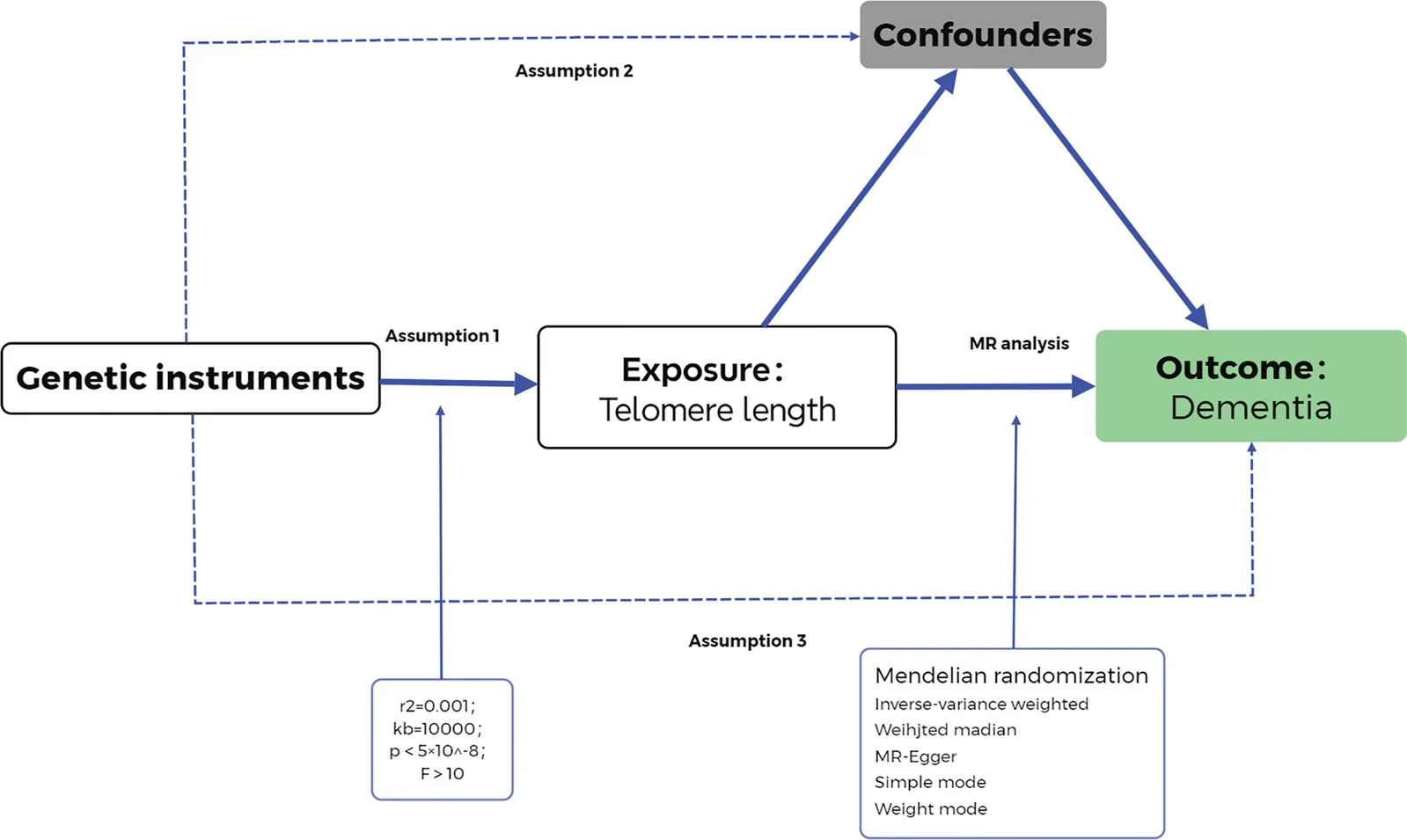
Flowchart of the 2-sample Mendelian randomization study design.

Two-sample Mendelian randomization analysis was used to determine whether there was a causal relationship between genetically predicted telomere length and dementia (including all-cause dementia, AD, VD, FTD, PD). An overview of the study design is shown in Figure 2. To study the causal effects of exposure on outcomes, the selection of IVs in MR analysis should satisfy the following 3 assumptions: there should be a strong correlation between IVs and telomere length; IVs are independent of confounders in the exposure-outcome causality; and IVs affect dementia incidence only through telomere length.

#### 2.3.2. Data sources

MR analysis was performed using publicly available pooled data from the GWAS database (Table S1, Supplemental Digital Content, https://links.lww.com/MD/R604). To determine exposure factors, genetic data on telomere length (N = 472,174) from the IEU OpenGwas database^[24]^ were utilized. For outcome factors, genetic data on all-cause dementia (4,94,845 participants, 24,864 cases, 4,69,981 controls), AD (4,77,949 participants, 7968 cases, 4,69,981 controls), VD (4,79,108 participants, 3624 cases, 4,75,484 controls), FTD (4,74,879 participants, 177 cases, 4,74,702 controls) and PD (5,00,348 participants, 5861 cases, 4,94,487 controls) were used from the FinnGen public availability.^[25]^ The data utilized in the MR analysis are exclusively from European descent, to mitigate potential bias arising from disparate racial backgrounds. The requirement for ethical approval or patient consent does not apply to this study, as the data are published in the public domain.

#### 2.3.3. Instrumental variables selection

To ensure the reliability and robustness of MR analysis results, we formulated the following rules for the selection of IVs: single-nucleotide polymorphisms (SNPs) as IVs need to show strong correlation with telomere length (*P* < 10^–8^, *F* ≥** **10), *F* statistic is calculated using the formula: *F* = (*R*^2^/(1 − *R*^2^)) × ((N − *K* − 1)/*K*), *R*^2^ is calculated using the formula: *R*^2^ = 2 × *MAF* × (1 − *MAF*) × β^2[26]^; to reduce potential bias due to strong linkage disequilibrium between SNPs, only IVs with low association with other SNPs were retained, defined as *R*^2^ < 0.001 within a 10,000 kb window; linkage disequilibriumTrait tool^[27]^ to remove SNPs associated with the outcome or key confounders. Additionally, before analysis, we performed strict IV harmonization: all ambiguous SNPs with unclear direction were directly excluded, completely eliminating the risk of bias due to allele confounding.

#### 2.3.4. Statistical analysis

The primary method of obtaining causality estimates was via inverse variance weighting, with additional methods employed including MR-Egger and weighted median methods. A *P*-value of <.05 was considered to indicate a significant causal relationship. To visualize the causal relationships between exposure factors and outcomes, the odds ratio values were transformed into the beta coefficient (β) as follows: β = ln [OR]. In order to ensure the reliability and robustness of the study, a variety of methods were used to conduct sensitivity analysis. The heterogeneity between the IVs was assessed using Cochran’s *Q* test. The sensitivity of individual SNPs was assessed by the leave-one-out plot. The analysis, employing a funnel plot, revealed an asymmetry in effect estimation. The MR-Egger intercept test was utilized to elucidate pleiotropic effects. For interpretation purposes, a *P*-value < .05 in the Cochran *Q* test or the MR-Egger intercept test is considered to indicate heterogeneity or horizontal pleiotropy.^[28]^ All data analysis was performed using R software version 4.4.3.

## 3. Results

### 3.1. Cross-sectional study by NHANES

The analysis was based on the NHANES sample weights, and the sample analyzed in this study represented approximately 27 million non-institutional participants in the United States. Participant characteristics stratified by telomere length tertile are presented in Table 1. In weighted multivariate linear regression models (Table 2), longer telomere length was consistently associated with higher cognitive function scores across progressively adjusted models.

**Table 1:** Baseline characteristics of the United States adults aged 60 years and older by telomere length tertiles in the National Health and Nutrition Examination Survey, 1999–2002

Characteristics	Mean leukocyte telomere length (T/S ratio)				
	Overall	T1 ≤ 0.801	T2 0.801–0.963	T3 ≥ 0.963	*P*-value
n	2,69,97,239	90,00,743	90,18,063	89,78,433	
DSST mean (SD)	48.05 (16.87)	44.40 (16.83)	48.43 (16.00)	51.34 (17.07)	<.001
Age (%)					<.001
60–69	1,49,04,215 (55.2)	37,20,201 (41.3)	51,32,032 (56.9)	60,51,982 (67.4)	
>70	1,20,93,024 (44.8)	52,80,542 (58.7)	38,86,031 (43.1)	29,26,451 (32.6)	
Sex (%)					.013
Male	1,22,31,981 (45.3)	43,05,306 (47.8)	43,69,412 (48.5)	35,57,263 (39.6)	
Female	1,47,65,258 (54.7)	46,95,437 (52.2)	46,48,651 (51.5)	54,21,170 (60.4)	
Ethnicity (%)					.819
Non-Hispanic White	2,29,59,708 (85.0)	76,36,524 (84.8)	77,70,813 (86.2)	75,52,371 (84.1)	
Non-Hispanic Black	15,52,005 (5.7)	4,71,256 (5.2)	4,91,085 (5.4)	5,89,664 (6.6)	
Mexican American	7,20,582 (2.7)	2,57,085 (2.9)	2,20,390 (2.4)	2,43,107 (2.7)	
Other Hispanic	11,11,864 (4.1)	3,73,156 (4.1)	3,01,932 (3.3)	4,36,776 (4.9)	
Other race	6,53,080 (2.4)	2,62,722 (2.9)	2,33,843 (2.6)	1,56,515 (1.7)	
Educational level (%)					.863
High school or less	1,54,12,345 (57.1)	52,14,036 (57.9)	51,40,141 (57.0)	50,58,168 (56.3)	
College Graduate or above	1,15,84,894 (42.9)	37,86,707 (42.1)	38,77,922 (43.0)	39,20,265 (43.7)	
Poor income ratio (%)					.017
<1	26,64,907 (9.9)	8,76,310 (9.7)	10,64,994 (11.8)	7,23,603 (8.1)	
1–1.99	72,19,848 (26.7)	28,84,818 (32.0)	23,33,601 (25.9)	20,01,429 (22.3)	
2–3.99	87,82,848 (32.5)	27,54,930 (30.6)	30,72,202 (34.1)	29,55,716 (32.9)	
>4	83,29,636 (30.9)	24,84,685 (27.6)	25,47,266 (28.2)	32,97,685 (36.7)	
Physical activity level (%)					.277
Very low	64,59,574 (23.9)	25,42,021 (28.2)	19,18,057 (21.3)	1,99,94,96 (22.3)	
Low	1,61,48,637 (59.8)	52,16,502 (58.0)	56,33,319 (62.5)	52,98,816 (59.0)	
Medium	3,89,02,94 (14.4)	10,93,517 (12.1)	12,90,436 (14.3)	15,06,341 (16.8)	
High	4,98,734 (1.8)	1,48,703 (1.7)	1,76,251 (2.0)	1,73,780 (1.9)	
Smoking status (%)					.515
Never	1,25,48,666 (46.5)	40,94,927 (45.5)	40,15,718 (44.5)	44,38,021 (49.4)	
Former	1,11,26,365 (41.2)	37,06,708 (41.2)	38,28,981 (42.5)	35,90,676 (40.0)	
Current	3,322,208 (12.3)	11,99,108 (13.3)	11,73,364 (13.0)	9,49,736 (10.6)	
Alcohol status (%)					.351
No	1,03,96,029 (38.5)	37,16,281 (41.3)	35,03,394 (38.8)	31,76,354 (35.4)	
Yes	16,601,210 (61.5)	5284,462 (58.7)	5514,669 (61.2)	58,02,079 (64.6)	
Self-reported diseases (%)					
Hypertension	13,479,625 (49.9)	46,36,991 (51.5)	41,03,359 (45.5)	47,39,275 (52.8)	.035
Diabetes mellitus	38,18,220 (14.1)	14,36,624 (16.0)	13,13,785 (14.6)	10,67,811 (11.9)	.321
Congestive heart failure	15,63,627 (5.8)	6,21,437 (6.9)	6,01,812 (6.7)	3,40,378 (3.8)	.134
Stroke	14,73,074 (5.5)	5,77,514 (6.4)	4,94,074 (5.5)	4,01,486 (4.5)	.406
Cancer or malignancy	56,11,912 (20.8)	20,08,196 (22.3)	18,86,817 (20.9)	17,16,899 (19.1)	.462
Body mass index (%)					.936
Underweight	4,13,207 (1.5)	1,52,456 (1.7)	1,23,470 (1.4)	1,37,281 (1.5)	
Normal	74,93,878 (27.8)	25,49,768 (28.3)	25,54,688 (28.3)	23,89,422 (26.6)	
Overweigh	1,05,37,798 (39.0)	36,26,804 (40.3)	34,37,110 (38.1)	34,73,884 (38.7)	
Obese	85,52,356 (31.7)	26,71,715 (29.7)	29,02,795 (32.2)	29,77,846 (33.2)	

All estimates accounted for sample weights and complex survey designs and means and percentages were adjusted for survey weights of NHANES.

DSST = digit symbol substitution test, SD = standard deviation, T/S ratio = telomere/single-copy gene.

**Table 2:** Multiple linear regression analysis of the association between telomere length and Digit Symbol Substitution Test scores in the National Health and Nutrition Examination Survey, 1999–2002.

Characteristic	Mean leukocyte telomere length (T/S ratio)			*P*-trend
	T1 ≤ 0.801	T2 0.801–0.963	T3 ≥ 0.963	
Model 1	Ref.	4.0 (1.70–6.3)**	6.9 (4.7–9.1)***	<.001
Model 2	Ref.	2.3 (0.15–4.5)*	3.9 (1.8–6.0)***	<.001
Model 3	Ref.	2.1 (0.35–3.9)*	2.5 (0.69–4.3)*	.004

All values represented are OR (95% CI). Data are weighted according to the NHANES protocol.

Model 1: unadjusted model.

Model 2: adjusted for age, sex, and ethnicity.

Model 3: further adjusted for BMI, alcohol status, smoking status, educational level, poor income ratio, physical activity level and self-reported diseases (hypertension, diabetes mellitus, congestive heart failure, stroke and cancer or malignancy).

BMI = body mass index, CI = confidence interval, NHANES = National Health and Nutrition Examination Survey, OR = odds ratio.

* *P* < .05.

** *P* < .01.

*** *P* < .001.

**Figure 3.**
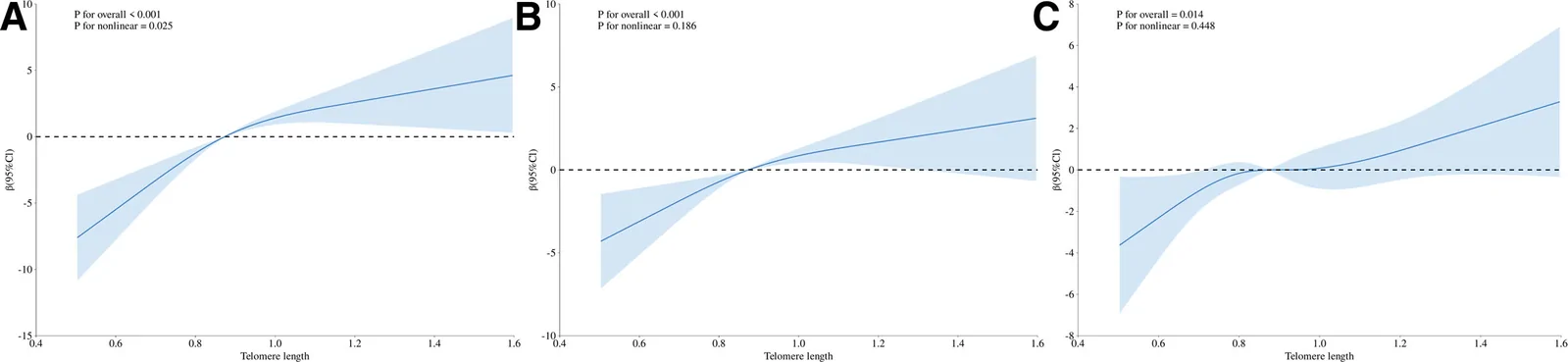
Restricted cubic spline curves showing the association between telomere length and digit symbol substitution test scores. Panels A–C correspond to model 1 to 3, respectively. Model 1 was unadjusted; model 2 was adjusted for age, sex, and ethnicity; model 3 was further adjusted for BMI, alcohol status, smoking status, educational level, poor income ratio, physical activity level and self-reported diseases (hypertension, diabetes mellitus, congestive heart failure, stroke and cancer or malignancy). BMI = body mass index.

The Restricted cubic spline model was used to examine the shape of the association (Fig. 3A: model 1; Fig. 3B: model 2; Fig. 3C: model 3). After adjustment for all covariates (Fig. 3C), it suggested a potential non-linear relationship. However, the test for non-linearity was not statistically significant (*P* = .448). Accordingly, a linear model provides an adequate representation of the association between telomere length and cognitive function in this cohort.

### 3.2. MR analysis

**Figure 4.**
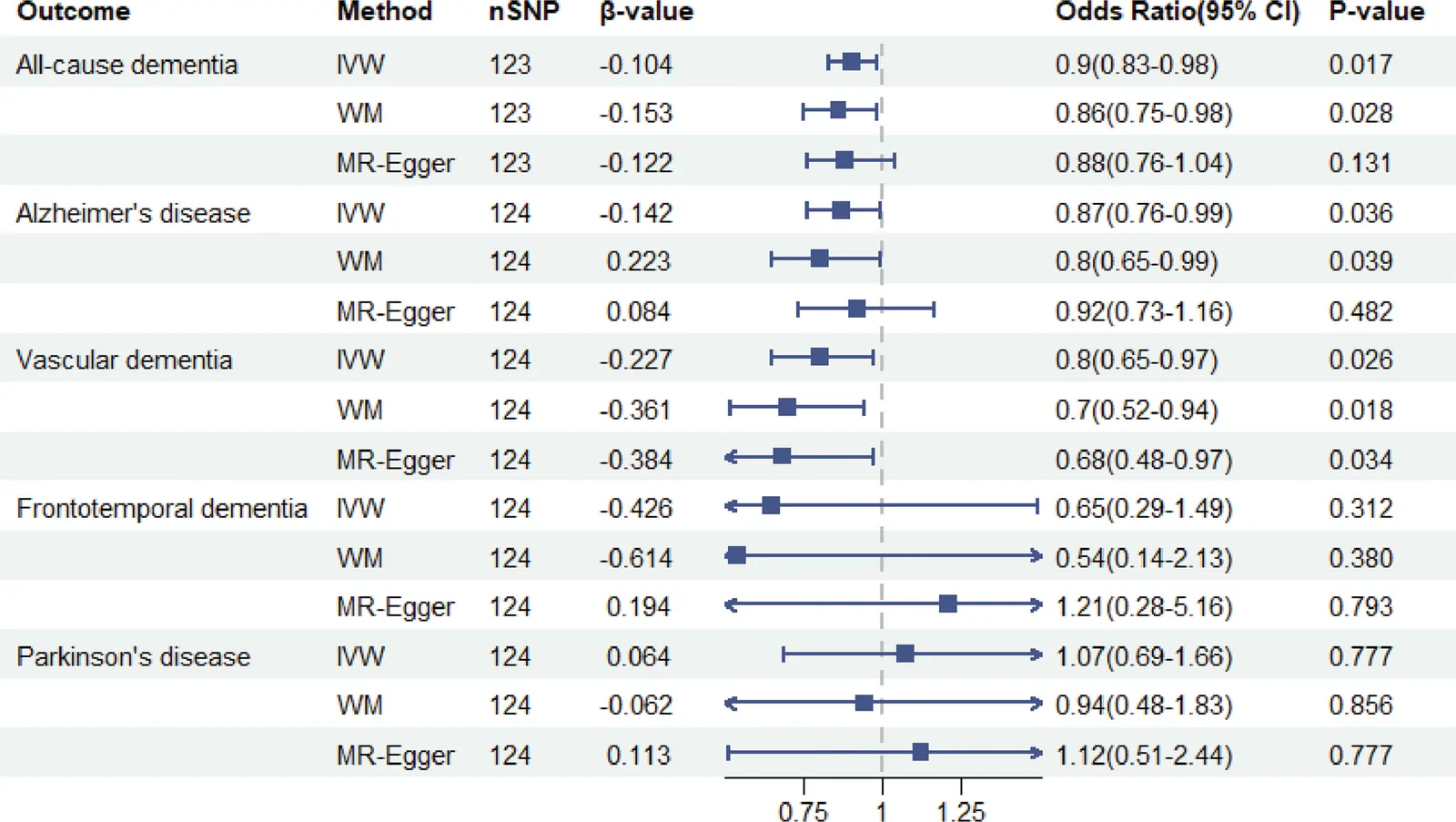
Causal associations between genetically predicted telomere length and dementia risk estimated using Mendelian randomization. CI = confidence interval, SNP = single-nucleotide polymorphism.

**Figure 5.**
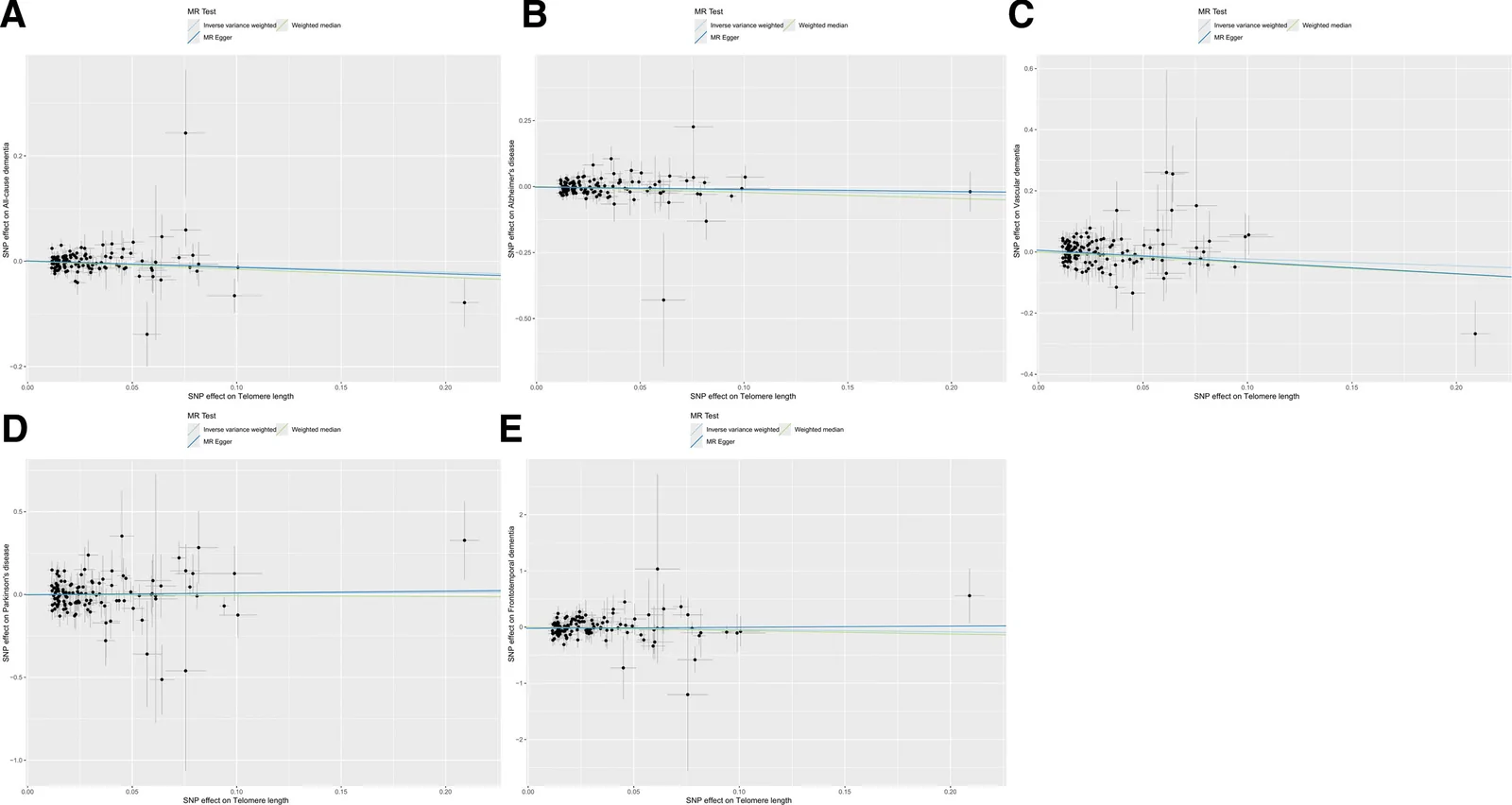
Scatter plots illustrating the causal associations between telomere length and dementia risk using Mendelian randomization. (A) All-cause dementia; (B) Alzheimer's disease; (C) Vascular dementia; (D) Parkinson's disease; (E) Frontotemporal dementia.

As shown in Figures 4 and 5, genetically predicted longer telomere length was causally associated with a reduced risk of all-cause dementia based on inverse variance weighting analysis. Significant protective associations were also observed for AD and VD, but not for FTD or PD.

These causal inferences were based on strong genetic instruments (mean *F*-statistic = 116.16, range: 29.89–1633.59). To ensure the reliability and robustness of the study, we performed a series of sensitivity analyses. The p-values of the Cochran *Q* test and the MR-Egger intercept test were both >0.05 (Table S2, Supplemental Digital Content, https://links.lww.com/MD/R604), suggesting no substantial heterogeneity or horizontal pleiotropy among the selected IVs. The results of the leave-one-out Plot (Fig. S1, Supplemental Digital Content, https://links.lww.com/MD/R604) and the funnel plot (Fig. S2, Supplemental Digital Content, https://links.lww.com/MD/R604) indicated that no single genetic variant drove the results and showed no asymmetry.

## 4. Discussions

Based on NHANES data combined with Mendelian randomization studies, this study clarified a positive correlation between telomere length and cognitive function through the analysis of a large amount of data at the clinical level. Mendelian randomized studies have shown that shortening of telomere length is positively associated with the development of all-cause dementia, VD, and AD. However, no such correlation has been observed about FTD and PD.

Telomeres are repetitive DNA sequences located at the ends of chromosomes, and progressive telomere shortening is a key driver of cellular senescence and apoptosis.^[29-31]^ In somatic cells, including neurons, replicative senescence is accompanied by suppressed telomerase activity, leading to impaired telomere maintenance and apoptosis once telomere length falls below a critical threshold.^[32]^ In the nervous system, this process may lead to extensive neuronal loss, thereby triggering cognitive dysfunction.^[33]^ Consistently, experimental studies have shown that reactivation of telomerase in telomerase-deficient mice can reverse several features of brain aging, including neural stem cell proliferation, brain tissue integrity, and olfactory function,^[34]^ providing mechanistic support for the involvement of telomere maintenance in brain aging.

In addition, telomere dysfunction has been associated with an accelerated release of pro-inflammatory cytokines, such as IL-6, TNF-α and IL-1β, thereby promoting an inflammatory microenvironment.^[35]^ Neuroinflammation has been demonstrated to exacerbate amyloid-β accumulation and tau hyperphosphorylation, 2 central pathological processes in the onset and progression of AD.^[36]^ These mechanisms align with our MR results, providing genetic support for a causal link between shorter telomere length and AD risk.

In summary, it can be concluded that shortened telomere length may jointly promote the development of cognitive dysfunction and dementia by leading to cellular senescence, death, and chronic inflammation of the nervous system.

Different from previous single studies, this study demonstrated the correlation between telomere length and cognitive function at both phenotypic and genetic levels, improving the reliability of the evidence. This study was based on a large sample size and covered multiple sub-types of dementia. Based on the NHANSE database, we observed that longer telomere length was associated with better cognitive function, and Mendelian randomization analysis further supported the protective role of longer telomere length for all-cause dementia and AD and VD, and that shorter telomere length plays a key role in the development of neurodegenerative diseases. Our study provides evidence linking telomere length of genetic agents to dementia risk, suggesting that exploring telomere-targeted interventions may be a new avenue for preventing neurodegenerative diseases in the future.

### 4.1. Limitations

Several limitations of this study should be acknowledged. First, the populations included in the cross-sectional analysis and the MR analysis were not identical. This inconsistency in the population ancestry may introduce heterogeneity and limits the direct comparability between the observational and genetic causal estimates. Second, a substantial proportion of initially eligible NHANES participants were excluded due to missing data on cognitive assessments, telomere length measurements, or relevant covariates. Such exclusions may have introduced selection bias and reduced the representativeness of the final analytical sample. Third, the generalizability of our findings may be limited, as both the cross-sectional and MR analyses were restricted to the US populations and populations of European ancestry, respectively, and may not be directly applicable to other ethnic or geographic populations.

Future studies are warranted to validate our findings and to clarify the biological mechanisms underlying the association between telomere length and neurodegenerative diseases. In particular, future research integrating large-scale, multi-omics approaches may help elucidate the molecular pathways through which telomere shortening contributes to cognitive decline and dementia. Such studies across diverse populations would also strengthen the generalizability of these findings.

## 5. Conclusions

In conclusion, this study demonstrated a positive correlation between telomere length and cognitive function through a cross-sectional study and Mendelian randomization study, and longer telomere length has a protective effect on all-cause dementia, AD and VD.

## Acknowledgments

Cross-sectional study data were from NHANES and MR study data from genome-wide association meta-analyses by IEU OpenGWAS and FinnGen Consortium. The authors thank all researchers for sharing these data.

## Author contributions

**Conceptualization:** Li Geng.

**Formal analysis:** Yuhang Shen.

**Funding acquisition:** Li Geng.

**Methodology:** Yuhang Shen.

**Software:** Yuhang Shen.

**Supervision:** Li Geng.

**Writing – original draft:** Yuhang Shen, Jingdong Zhou.

**Writing – review & editing:** Yuhang Shen, Jingdong Zhou, Bing Sun, Li Geng.

## Supplementary Material


