# Supplementary material for: Association of telomere length with cognitive function and dementia: A cross-sectional NHANES analysis and 2-sample Mendelian randomization study
Source: Medicine (Baltimore). 2026 Apr 10;105(15):e48217. doi: 10.1097/MD.0000000000048217 (PMC13593167; doi:10.1097/MD.0000000000048217)

Table S1. Mendelian Randomization data source of telomere length and dementia

Phenotype	Dataset	Total sample size	Case; control sample sizes
Telomere length	ieu-b-4879	472,174	NA
All-cause dementia	gs://finngen-public-data-r12/summary_stats/release/finngen_R12_F5_DEMENTIA.gz	494,845	24,864; 469,981
Alzheimer's disease	gs://finngen-public-data-r12/summary_stats/release/finngen_R12_F5_ALZHDEMENT.gz	477,949	7,968; 469,981
Vascular dementia	gs://finngen-public-data-r12/summary_stats/release/finngen_R12_F5_DEMENTIA.gz	494,845	24,864; 469,981
Frontotemporal dementia	gs://finngen-public-data-r12/summary_stats/release/finngen_R12_FTD.gz	474,879	177; 474,702
Parkinson's disease	gs://finngen-public-data-r12/summary_stats/release/finngen_R12_G6_PARKINSON.gz	500,348	5,861; 494,487

Abbreviation: NA, not applicable.

Table S2. Pleiotropy and heterogeneity tests for the causal link between telomere length and dementia risk

Outcome	Heterogeneity		Pleiotropy	
	Cochran's Q test		MR-Egger intercept test	
	IVW	MR-Egger		
	p-value	p-value	Intercept	p-value
All-cause dementia	0.342	0.320	6e-4	0.790
Alzheimer's disease	0.539	0.522	−0.002	0.548
Vascular dementia	0.167	0.171	0.006	0.288
Frontotemporal dementia	0.767	0.769	−0.022	0.308
Parkinson's disease	0.087	0.077	−0.002	0.881

Abbreviations: IVW, inverse variance weighting; MR, Mendelian randomization.

Figure S1. Leave-one-out sensitivity analyses for the Mendelian randomization estimates of telomere length on dementia risk

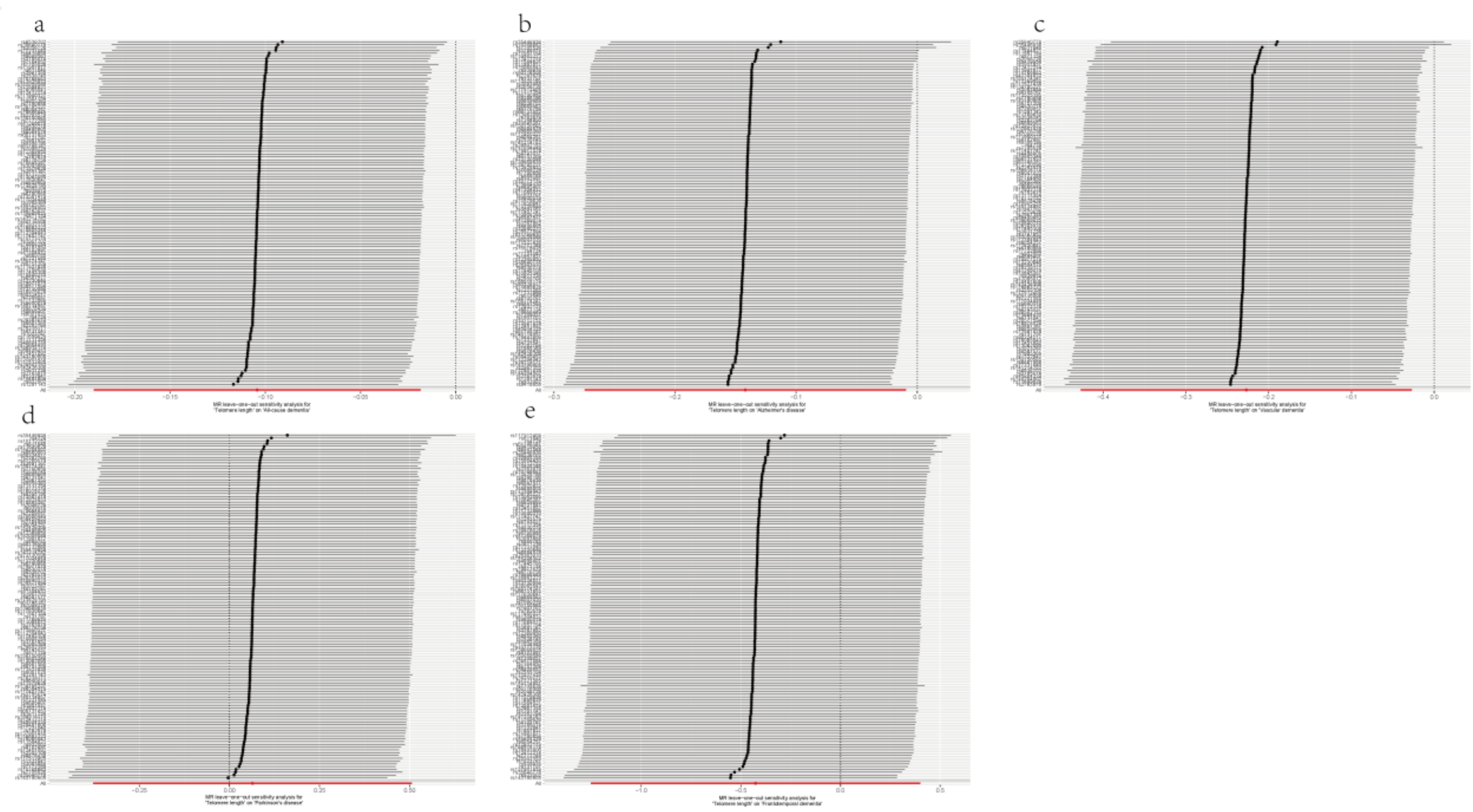

Figure S2. Funnel plots assessing heterogeneity and potential horizontal pleiotropy in the Mendelian randomization analyses of telomere length and dementia risk

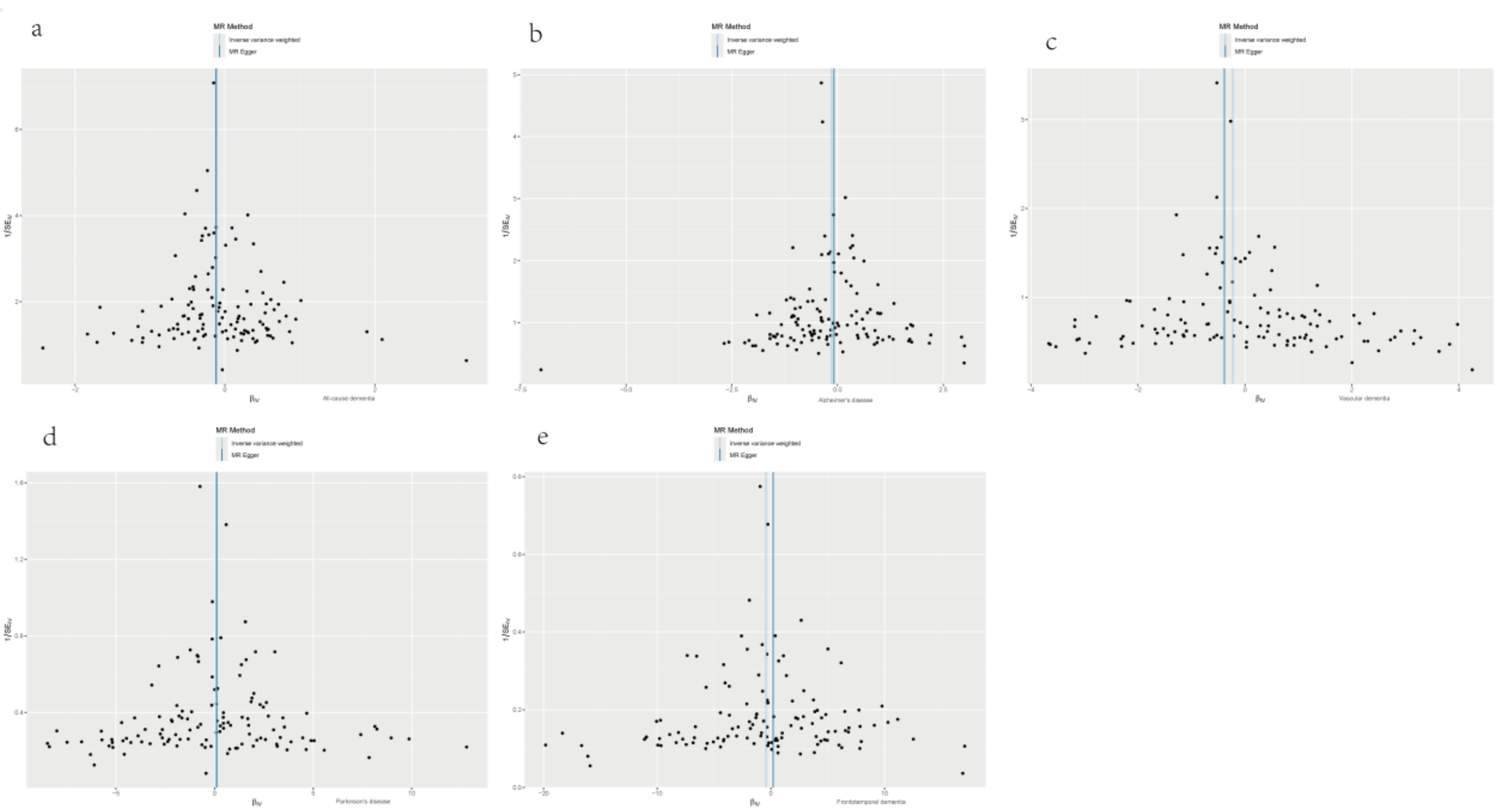

Supplement: Supplementary file 1 [file medi-105-e48217-s001.pdf]
